# Clinical Significance of *Fusobacterium nucleatum* Infection and *Regulatory T Cell* Enrichment in Esophageal Squamous Cell Carcinoma

**DOI:** 10.3389/pore.2021.1609846

**Published:** 2021-07-09

**Authors:** Ning Zhang, Yiwen Liu, Hong Yang, Mengxia Liang, Xiaopeng Wang, Min Wang, Jinyu Kong, Xiang Yuan, Fuyou Zhou

**Affiliations:** ^1^ Anyang Tumor Hospital, The Fourth Affiliated Hospital of Henan University of Science and Technology, Anyang, China; ^2^ Henan Key Laboratory of Cancer Epigenetics, Cancer Institute, The First Affiliated Hospital, College of Clinical Medicine, Henan University of Science and Technology, Luoyang, China; ^3^ School of PE, Henan University of Science and Technology, Luoyang, China

**Keywords:** Esophageal squamous cell carcinoma, *Fusobacterium* nucleatum, Prognostic value, regulatory T cells, clinical significance

## Abstract

A variety of pathogenic microorganisms promote tumor occurrence and development through long-term colonization in the body. *Fusobacterium nucleatum* (*F. nucleatum*) is abundant in precancerous esophageal lesions and is closely related to the malignant progression of esophageal squamous cell carcinoma (ESCC). The invasion of exogenous microorganisms can reshape the immune microenvironment, make the immune system incapacitated, and assist tumor cells in immune escape. A variety of pathogenic microorganisms induce the recruitment of *regulatory T cell* (*Tregs*) to allow tumor cells to escape immune surveillance and provide favorable conditions for their own long-term colonization. *Tregs* are one of the major obstacles to tumor immunotherapy and have a significant positive correlation with the occurrence and development of many kinds of tumors. Because *F. nucleatum* can instantly enter cells and colonize for a long time, we speculated that F. nucleatum infection could facilitate the immune escape of tumor cells through enrichment of Tregs and promote the malignant progression of ESCC. In this study, we found a significant concordance between *F. nucleatum* infection and *Tregs* infiltration. Therefore, we propose the view that chronic infection of *F. nucleatum* may provide favorable conditions for long-term colonization of itself by recruiting *Tregs* and suppressing the immune response. At the same time, the massive enrichment of *Treg* may also weaken the immune response and assist in the long-term colonization of *F. nucleatum*. We analyzed the correlation between *F. nucleatum* infection with the clinicopathological characteristics and survival prognosis of the patients. *F. nucleatum* infection was found to be closely related to sex, smoking, drinking, degree of differentiation, depth of invasion, lymph node metastasis, and clinical stage. The degree of differentiation, depth of infiltration, lymph node metastasis, clinical stage, and *F. nucleatum* infection are independent risk factors affecting ESCC prognosis. Additionally, the survival rate and median survival time were significantly shortened in the *F. nucleatum* infection positive group. Therefore, we propose that long-term smoking and alcohol consumption cause poor oral and esophageal environments, thereby significantly increasing the risk of *F. nucleatum* infection. In turn, *F. nucleatum* infection and colonization may weaken the antitumor immune response through *Treg* enrichment and further assist in self-colonization, promoting the malignant progression of ESCC.

## Introduction

Esophageal cancer has very high morbidity and mortality rates[1]. There are approximately 500,000 new cases of esophageal cancer worldwide every year, more than half of which occur in China [2]. Squamous cell carcinoma is the most common histological type of esophageal cancer, accounting for more than 95% of all esophageal cancers. The prognosis of esophageal squamous cell carcinoma (ESCC) is extremely poor. Although traditional surgery, radiotherapy and chemotherapy, targeted therapy, and immunotherapy are constantly used in comprehensive tumor treatment, the 5 years survival rate of patients with advanced disease is still less than 20% [3]. The etiology of ESCC is not completely clear, and its risk factors include smoking, drinking, diet, chronic infection, immune dysfunction, and genetic susceptibility. Studies have shown that a variety of pathogenic microorganisms can promote the occurrence and development of tumors through long-term colonization in the body [4-6]. Although the mechanism of pathogenic microorganism infection in tumors is not completely clear, the removal of pathogenic microorganisms is helpful to control the malignant progression of tumors.

*Fusobacterium nucleatum* (*F. nucleatum*), a conditionally pathogenic bacterium that colonizes in the oral cavity, can cause a microecological imbalance by altering its population [7]. Due to the absence of venous valves in the oral and maxillofacial regions, *F. nucleatum* can easily spread throughout the body in the circulation, participate in the progression of many diseases [8], and is closely related to the occurrence and development of many tumors [9]. Studies have shown that *F. nucleatum* is abundant in precancerous esophageal lesions [10], and the survival time of patients with ESCC infected with *F. nucleatum* is significantly shortened [11]. Although the specific pathogenic mechanism is not clear, *F. nucleatum* is closely related to the malignant progression of ESCC. Previous studies on the relationship between *F. nucleatum* infection and ESCC have mostly focused on tumor cells themselves, but there is little discussion on the role of the tumor microenvironment. In fact, the occurrence and development of tumors is closely related to the tumor microenvironment [12-13]. The tumor microenvironment not only provides a rich material basis for the malignant behavior of the tumor but also regulates immune cells to prevent their normal function, leading to tumor immune escape and promoting malignant progression [14]. Immunosuppression mediated by *regulatory T cells* (*Tregs*) is not only one of the important mechanisms leading to the immune escape of tumor cells but also a key obstacle in tumor immunotherapy [15-16]. *Tregs*, a subset of *CD4* ^*+*^ *CD25* ^*+*^ *FoxP3* ^*+*^ *regulatory T cells* with immunosuppressive functions, play an important role in maintaining immune tolerance and regulating the level of the immune response [17] and are positively correlated with the malignant progression of tumors. Clinical data show that pathogenic microorganisms in a variety of tumors can recruit *Tregs* [18-21]. Therefore, it is hypothesized that *F. nucleatum* infection and *Treg* enrichment may assist in immune escape of tumor cells and promote malignant progression of ESCC.

In this study, RNAscope and immunohistochemistry (IHC) were used to detect *F. nucleatum* infection and *Tregs* infiltration, respectively, in ESCC tissues and to analyze *F. nucleatum* infection correlation with the clinicopathological features and survival prognosis of patients to provide new strategies and therapeutic tools for the treatment of ESCC.

## Materials and Methods

### Study Subjects

For this study, a total of 246 patients with ESCC from Anyang Tumor Hospital (ATH; Anyang, Henan, China) were enrolled after radical resection. Tumor tissue specimens and corresponding paracancerous tissue specimens (esophageal mucosa more than 5 cm from the edge of the cancer tissue) were collected from each patient for formalin fixation and paraffin embedding. The inclusion criteria were as follows: 1) patients did not receive radiotherapy, chemotherapy, or immunotherapy before the operation; 2) no antibiotics were used within four weeks prior to operation; 3) specimens were taken after therapeutic resection of ESCC; 4) postoperative pathological diagnosis of ESCC was performed; 5) comprehensive case information was provided; and 6) the follow-up period was 60 months (5 years). The exclusion criteria were as follows: 1) preoperative radiotherapy, chemotherapy, or immunotherapy; 2) postoperative pathological diagnosis that was not ESCC or complicated with other tumors; 3) received immunotherapy after the operation; 4) incomplete case information; and 5) death not caused by ESCC.

The institutional review boards of ATH reviewed and approved this study (No. 2020-05-B005), and all participants provided written informed consent. Patient information was anonymized and deidentified prior to analysis. Basic patient information, including age, sex, and tobacco and alcohol histories (Smokers were defined as those who smoked at least one cigarette per day and smoked regularly for at least 6 months continuously or cumulatively and were considered smoking positive; others were considered "never smokers" and were considered smoking negative [22]. Alcohol drinkers were defined as those who consumed at least 25 g of alcohol per day on average and were considered positive for alcohol consumption; others were considered "occasional drinkers or never drinkers" and were considered negative for alcohol consumption [23].), and clinical information, including the histological type, degree of tumor invasion and differentiation, lymph node metastasis, tumor-node-metastasis (TNM) stage, and clinical stage, were collected from hospital records. The clinical stage and histological type were based on the 2017 Union for International Cancer Control (UICC)/American Joint Committee on Cancer (AJCC) TNM classification system (eighth edition) for esophageal cancer. ATH has a standard follow-up center, and the patients or their families were contacted every 3 months until the end of follow-up. The end of follow-up was postoperative death, and the surviving patients were followed up to 60 months after the operation. If patients could not be contacted by telephone, they were visited at their homes in the countryside or were instructed to go to the local police station for confirmation.

### RNAscope and Scoring

The RNAscope® 2.5 High Definition (HD)-Red Assay is based on Advanced Cell Diagnostics, Inc. (ACD)’s patented signal amplification and background suppression technology. The assay uses a novel and proprietary method of *in situ* hybridization (ISH) to visualize single RNA molecules per cell in a multitude of sample types mounted on slides. The RNAscope® 2.5 HD (ACD) was used in accordance with the manufacturer’s instructions [22], with minor modifications (see below). The RNAscope assay was performed on formalin-fixed paraffin-embedded (FFPE) sections using the RNAscope 2.5 HD Assay-RED (322,350, ACD, United States). The paraffin-embedded specimens of ESCC tissues and corresponding paracancerous tissues from each patient were sectioned continuously at 3 μm. The specimens were immediately placed into xylene for dewaxing at 60°C for 1.5 h (2 × 5 min) and subjected to ethanol dehydration (2 × 1 min). RNAscope hydrogen peroxide (322,335, ACD, United States) was added and incubated for 10 min, the slides were placed in boiling (98–102°C) RNAscope target repair reagent (322,000, ACD, United States) for 15 min and immediately rinsed in distilled water (3 times), and hydrophobic circles were drawn when the slides were completely dry (room temperature). RNAscope Protease Plus (322,331, ACD, United States) was added and incubated for 30 min at 40°C in a HybEZ Hybridization furnace (220 VAC, 310,013, ACD, United States). The slides were then hybridized with an *F. nucleatum* probe (RNAscope® Probe—B-Fusobacterium-23S-3zz, Cat. No. 486411, ACD, United States) for 2 h at 40°C. After hybridization, slides were subjected to signal amplification using the RNAscope® 2.5 HD Detection Kit-RED (322,360, ACD, United States). The tissue sections that had been incubated with the probe were incubated with Amp 1 (preamplifier) for 30 min at 40°C, Amp 2 (background reducer) for 15 min at 40°C, Amp 3 (amplifier) at 40°C, Amp 4 (label probe) for 15 min at 40°C, Amp 5 for 30 min at room temperature, and finally Amp 6 for 15 min at room temperature. After each of these steps, the sections were rinsed in wash buffer (310,091, ACD, United States). Then, the hybridization signal was detected using a mixture of Fast-RED solutions A and B (1:60). After counterstaining with Gill's hematoxylin, slides were dried in a 60°C dry oven for 15 min and sealed with VectaMount (321,584, ACD, United States). The *F. nucleatum* signal was observed in tumor cells as red granules. As recommended by ACD, a semi-quantitative scoring approach was applied to evaluate the staining results [24]. RNAscope results were examined under a standard bright-field microscope at 20-40× magnification. We used the scoring system provided by the vendor, as follows: 0: negative, 0–1 dots/10 tumor cells at 40×; 1+: 1−3 dots/cell visible at 20−40× magnification; 2+: 4–10 dots/cell visible at 20−40× magnification; 3+: >10 dots/cell and <10% positive cells with dot clusters visible at 20× magnification; and 4+: >10 dots/cell and >10% positive cells with dot clusters visible at 20× magnification. For each slide, five areas containing the highest number of positive cells or dot clusters were selected. All tumor cells within each field were counted, and then the percentage of positive *F. nucleatum* mRNA signals was used as the final score. Image acquisition and analysis were performed using 3DHISTECH software (automatic digital slide scanner, Pannoramic® MIDI I, 3DHISTECH, HU) and Lucia G software (Laboratory Imaging, Prague, Czech Republic) for microscopic image analysis [25-26].

### IHC and Scoring

The paraffin-embedded ESCC tissues and corresponding paracancerous tissues from the same patients were fixed with formalin, embedded in paraffin, and serially sectioned at a thickness of 3 μm. After the wax was dissolved in the oven at 60°C for 1.5 h, the samples were immediately placed into xylene for dewaxing (3 times in total, 10 min each time). The samples were successively hydrated in 100%, 95%, 85%, and 70% ethanol for 5 min, followed by continuous rinsing with distilled water for 5 min. Antigen repair was performed at 94–98°C with citrate buffer solution (C1032, Solarbio, China) for 15 min to fully expose antigens. Then, peroxidase blocker was added for 10 min to reduce endogenous peroxidase activity. After rinsing with phosphate-buffered saline (PBS, P1002, Solarbio, China), goat serum was added to reduce non-specific binding, and background staining was performed for 30 min. Serial sections were incubated with *CD4* ^*−*^, *CD25* ^*−*^ *,* and *FoxP3*-specific primary antibodies (PBS dilution ratio 1:100, Abcam, United Kingdom) overnight at 4°C. The next day, after the samples were reheated for 1 h and rinsed with PBS (3 times × 5 min), biotin-labelled secondary antibodies were added and incubated at room temperature for 1 h, and the samples were washed with PBS 3 times × 5 min. The SP-9000 SPlink Detection Kit (Biotin-Streptavidin HRP Detection Systems, SP-9000, ZSGB-BIO, China) was used according to the manufacturer’s instructions. Then, peroxidase-conjugated streptavidin, a biotin-binding protein, was added for sealing at room temperature for 15 min. The samples were washed with PBS (3 times × 5 min) and stained with diaminobenzidine tetrahydrochloride (DAB, DA1010, Solarbio, China) for color detection of antigen-antibody binding. The color was observed under a binocular stereomicroscope (Eclipse 80i, Nikon, Japan), and then the reaction was terminated with distilled water; then, hematoxylin redyeing was performed. The samples were subjected to gradient ethanol dehydration (70%, 85%, 95%, 100%) for 1 min each and then cleared with xylene (3 times × 3 min). After air drying, the samples were sealed with neutral gum. The expression of *CD4*, *CD25,* and *FoxP3* in ESCC tissue and corresponding paracancerous tissue sections was observed by optical microscopy in five randomly selected fields at 400x magnification (Eclipse 80i, Nikon, Japan). The positive sections jointly determined by two senior pathologists were taken as positive controls, and the negative sections (PBS was used instead of the primary antibody) were taken as negative controls. Conflicting results were resolved with a multihead microscope (Eclipse 80i, Nikon, Japan). Samples with light yellow, tan, or brown coloring in the same location of the lymphocyte membrane (*CD4* and *CD25*) or nucleus (*FoxP3*) in successive sections were considered positive for *Tregs* infiltration. A semi-quantitative scoring scheme based upon the average density of positive-staining cells for the section was utilized for assessment of expression of each immune marker. Five high-power microscopic fields (400 times) in the tissue sections were randomly selected for observation. Each tissue slide was evaluated by two senior pathologists. Staining was scored based on the number of positive-staining cells per 400x field with 1-5 cells designated as 1+, 6–30 cells as 2+, >30 cells as 3+, and no positive-staining cells as 0 [27]. A final score of ≥1 on all three slides of each patient was defined as positive for *Tregs* infiltration.

### Statistical Analysis

All statistical analyses were performed with the SPSS statistical package, version 26.0 (SPSS Inc., Chicago, IL, United States): 1) the correlation analysis was performed with the chi-square test, 2) the survival curve was drawn with the Kaplan-Meier method, 3) the difference between survival times was analyzed by the log-rank test, 4) the effect of factors on prognosis was analyzed using Cox regression, and 5) a *p* value < 0.05 was considered statistically significant.

## Results

### *F. nucleatum* Infection and *Treg* Enrichment in ESCC Tissues and Corresponding Paracancerous Tissues

**FIGURE 1:**
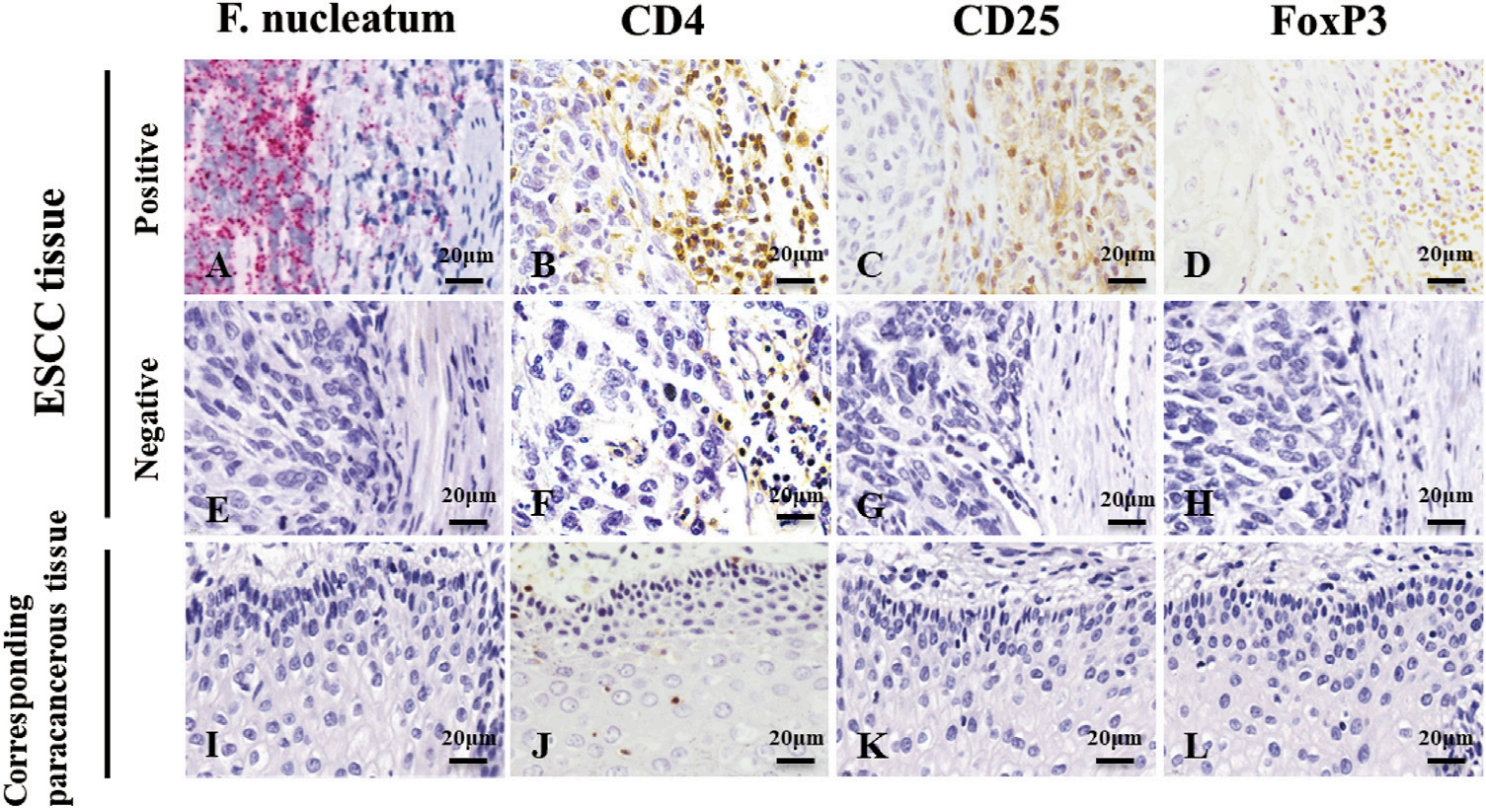
RNAscope detection image and IHC detection image. **(A,E,I)** are images of *F. nucleatum* in ESCC tissues and corresponding paracancerous tissues, while **(B,C,D,F,G,H,J,K,L)** are images of *Tregs* in ESCC tissues and corresponding paracancerous tissues. Red granules can be seen in the cytoplasm of ESCC tissue, indicating *F. nucleatum* infection **(A)**. In the serial section on the right side, light yellow, tan, or brown coloring was seen on the membrane (*CD4* and *CD25*) or nucleus (*FoxP3*) of lymphocytes at the same location, indicating infiltration of *Tregs* **(B,C,D)**. However, most cells were negative in the corresponding paracancerous tissues **(I,J,K,L)**. Original magnification 400×.

We used RNAscope and IHC to detect *F. nucleatum* infection and *Treg* enrichment in ESCC tissues and corresponding paracancerous tissues (Figure 1) and analyzed the correlation between the two factors. Red granules can be seen in the cytoplasm of ESCC tissues, indicating *F. nucleatum* infection (Figure 1A). In the serial sections, light yellow, tan, or brown coloring was seen on the membrane (*CD4* and *CD25*) or nucleus (*FoxP3*) of lymphocytes at the same location, indicating the positive infiltration of *Tregs* (Figure 1C,D). *F. nucleatum* infection was significantly correlated with *Tregs* infiltration (Table 1, *p* < 0.05). However, most cells in the corresponding paracancerous tissues were negative (Figure 1I,J,K,L). *F. nucleatum* infection in ESCC tissues was significantly higher than that in the corresponding paracancerous tissues (*p* < 0.05, Table 2). *Tregs* infiltration in ESCC tissues was significantly higher than that in the corresponding paracancerous tissues (*p* < 0.05, Table 3).

**TABLE 1:** Correlation between *F. nucleatum* infection and *Treg* infiltration in ESCC (chi-squared test).

		*F. nucleatum*		χ^2^	*P*
		Positive	Negative		
***Tregs***	Positive	75(97.40%)	2(2.60%)	195.782	0.01
	Negative	10(5.90%)	159(94.10%)		

**TABLE 2:** Comparison of the positive rate of *F. nucleatum* infection in ESCC tissues and corresponding paracancerous tissues in patients with ESCC.

		Carcinoma tissues		χ^2^	*P*
		*F. nucleatum* positive	*F. nucleatum* negative		
**Corresponding paracancerous tissues**	*F. nucleatum* positive	8(100%)	0(0%)	15.662	0.01
	*F. nucleatum* negative	77(32.35%)	161(67.65%)		

**TABLE 3:** Comparison of *Tregs* infiltration between ESCC tissues and corresponding paracancerous tissues in patients with ESCC.

		Carcinoma tissues		χ^2^	*P*
		*Tregs* positive	*Tregs* negative		
**Corresponding paracancerous tissues**	*Tregs* positive	7(100%)	0(0%)	15.662	0.01
	*Tregs* negative	70(29.29%)	169(70.71%)		

### Correlation of *F. nucleatum* Infection With the Clinicopathological Features of Patients With ESCC

Among the patients with *F. nucleatum* infection (Table 4), there were significantly more males, smokers, and drinkers (*p* < 0.05), and most of the tumor cells in the positive group were hypodifferentiated, with deeper tumor tissue infiltration, significantly more lymph node metastases, and more frequent occurrences of clinical stages III/IV (*p* < 0.05).

**TABLE 4:** Correlation between *F. nucleatum* infection and clinicopathological characteristics of patients with ESCC.

Factors	n	*F. nucleatum*		χ^2^	*P*
		Positive	Negative		
**Sex**					
Male	163	73(44.79)	90(55.21)	22.368	0.001
Female	83	12(14.46)	71(85.54)		
**Age (years)**					
≥60	139	42(30.22)	97(70.29)	2.658	0.103
<60	107	43(40.19)	64(59.81)		
**Smoking**					
Positive	125	73(58.40)	52(41.60)	63.908	0.001
Negative	121	12(9.92)	109(90.08)		
**Alcohol**					
Positive	119	73(61.34)	46(38.66)	73.165	0.001
Negative	127	12(9.45)	115(90.55)		
**Differentiation type**					
Poorly differentiated	52	36(69.23)	16(30.77)	35.063	0.001
Moderately-well differentiated	194	49(25.26)	145(74.74)		
**Infiltration depth**					
≥Adventitia	169	81(47.93)	88(52.07)	42.719	0.001
<Adventitia	77	4(5.19)	73(94.81)		
**Lymph node metastasis**					
Positive	107	81(75.70)	26(24.30)	141.785	0.001
Negative	139	4(2.88)	135(97.12)		
**Clinical stages**					
I/II	151	4(2.65)	147(97.35)	175.995	0.001
III/IV	95	81(85.26)	14(14.74)		

### Correlation of *F. nucleatum* Infection With Survival Prognosis in Patients With ESCC

**FIGURE 2:**
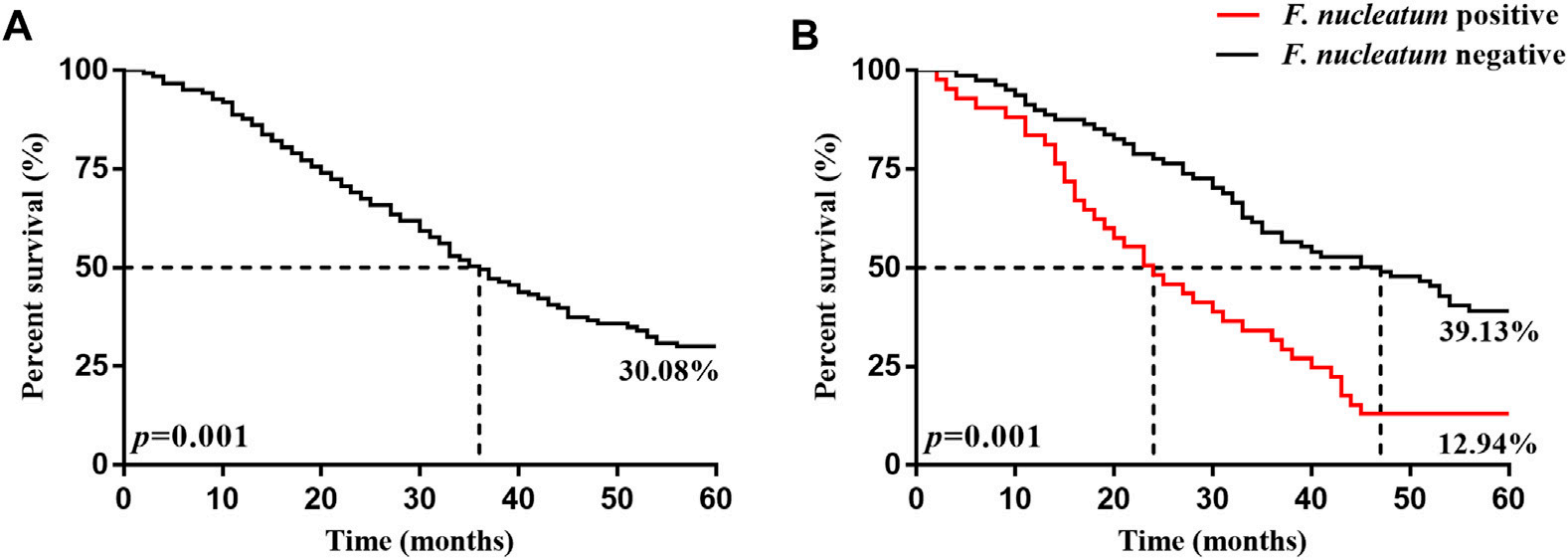
Kaplan-Meier 5 years survival curves of patients with ESCC patients 5 years after surgery. **(A)** is the 5 years survival curve of patients with ESCC patients. **(B)** is the 5 years survival curve of *F. nucleatum*-positive and negative patients after surgery.

The 5 years survival rate and median survival time of the 246 patients with ESCC after surgery were 30.08% and 36.0 months, respectively. The 5 years survival rate and median survival time in the *F. nucleatum* infection-positive group (12.94% and 47.0 months, respectively) were significantly lower (*p* < 0.05) than those in the negative group (39.13% and 24.0 months, respectively), as presented in Table 5 and Figure 2.

**TABLE 5:** Mean and median survival times (months) of patients with ESCC with *F. nucleatum* infection.

Group		Mean^a^				Median^a^				$\chi^{2}$	*P*
		Est	Std. Error	95% confidence interval		Est	Std. Error	95% confidence interval			
				Lower bound	Upper bound			Lower bound	Upper bound		
***F. nucleatum***	Positive	27.906	1.844	24.292	31.520	24.000	2.764	18.582	55.703	32.294	0.001
	Negative	41.839	1.455	38.987	44.690	47.000	4.440	38.297	51.970		
	Overall	37.024	1.221	34.631	39.418	36.000	2.091	31.901	40.099		

a Estimation was limited to the longest survival time; “Est.” and “Std.” represent “estimated” and “standard”, respectively.

### Cox Regression Analysis of Factors Affecting the Prognosis of Patients With ESCC

A total of 246 patients with ESCC successfully completed postoperative follow-up, with the death due to ESCC as the endpoint event, and univariate Cox regression analysis was conducted, *F. nucleatum* infection (positive = 1/negative = 0), sex (male = 1/female = 0), age (≥60 = 1/<60 = 0), smoking (positive = 1/negative = 0), alcohol consumption (positive = 1/negative = 0), degree of tumor differentiation (poor = 1/moderate/high = 0), depth of infiltration (≥adventitia = 1/<adventitia = 0), lymph node metastasis (positive = 1/negative = 0), and clinical stage (III/IV = 1/I/II = 0) were used as independent variables. The data showed that sex, smoking, alcohol consumption, degree of tumor differentiation, depth of infiltration, lymph node metastasis, clinical stage, and *F. nucleatum* infection were associated with overall survival (*p* < 0.05). Confounding factors (sex and age) were included, and variables that were significantly associated (*p* < 0.05) with ESCC in the univariate analysis were entered into multivariate Cox regression analysis, which showed that the degree of tumor differentiation, depth of infiltration, lymph node metastasis, clinical stage, and *F. nucleatum* infection are independent risk factors that affect the prognosis of ESCC (*p* < 0.05), as presented in Table 6.

**TABLE 6:** Cox regression analysis of prognostic factors in patients with ESCC.

Clinical variables	B	Wald	Hr	95%CI	*P*
**Univariate cox analysis**
Sex (male/Female)	0.743	17.888	2.103	1.490–2.968	0.001
Age (≥60/<60)	0.030	0.038	1.030	0.763–1.392	0.845
Smoking (positive/Negative)	1.221	54.697	3.392	2.454–4.689	0.001
Alcohol (positive/Negative)	1.267	60.030	3.549	2.576–4.890	0.001
Differentiation type (poorly/Moderately-Well)	0.837	22.507	2.309	1.634–3.263	0.001
Infiltration depth (≥Adventitia/<Adventitia)	0.982	26.969	2.670	1.843–3.869	0.001
Lymph node metastasis (positive/Negative)	1.040	44.511	2.828	2.084–3.839	0.001
Clinical stages (III/IV/I/II)	0.933	36.389	2.543	1.878–3.444	0.001
F. nucleatum (positive/Negative)	0.856	29.868	2.353	1.731–3.199	0.001

**Multivariate cox analysis**					
Sex (male/Female)	0.166	0.274	1.181	0.634–2.199	0.601
Age (≥60/<60)	0.070	0.140	1.072	0.744–1.546	0.709
Smoking (positive/Negative)	1.329	2.146	3.778	0.638–22.360	0.143
Alcohol (positive/Negative)	0.071	0.018	1.073	0.387–2.976	0.892
Differentiation type (poorly/Moderately-Well)	1.453	33.644	4.274	2.616–6.982	0.001
Infiltration depth (≥Adventitia/<Adventitia)	0.977	11.594	2.656	1.514–4.661	0.001
Lymph node metastasis (positive/Negative)	1.545	15.631	3.169	1.243–8.084	0.016
Clinical stages (III/IV/I/II)	1.154	5.830	4.222	1.105–16.134	0.035
F. nucleatum (positive/Negative)	2.861	11.542	17.478	3.355–91.049	0.001

## Discussion

ESCC has high morbidity and mortality rates and an extremely poor prognosis, and it is difficult to diagnose early. Therefore, it is particularly important to identify accurate early indicators, effective preventive measures, and targeted therapeutic approaches. For a long time, oncological research on chronic infection by pathogenic microorganisms has been lacking. In fact, a variety of pathogenic microorganisms can cause long-term colonization in tumor cells by reshaping the host immune microenvironment, leading to tumor immune escape and promoting malignant progression [28]. For *F. nucleatum*, one of the most virulent oral pathogenic bacteria [29], its endotoxin can suppress the body's immune response, thus colonizing the body for a long time and promoting the malignant progression of many tumors, such as oral squamous and esophageal squamous cancers and colon cancer [30].

The mechanism by which the tumor microenvironment is remodeled by pathogenic microorganisms has not been fully understood thus far, but most tumor microenvironments can recruit *Tregs* to deactivate the body's anti-tumor immunity through intercellular suppression mechanisms as well as through the secretion of immunosuppressive molecules, thus assisting in long-term colonization and the immune escape of tumor cells [31, 32]. The number of *Tregs* in the gastric mucosa of patients with *H. pylori* infection is significantly higher than that in the normal population, leaving the organism in an immunosuppressed state [33] and promoting the malignant proliferation of cancer cells. Cancer cells in patients with hepatitis B virus infection-positive hepatocellular carcinoma can induce cancer cell immune escape by upregulating the TGF-β protein and recruiting *Tregs* in large numbers [34]. Cancer cells in Epstein-Barr virus (EBV) infection-positive Hodgkin's lymphoma patients can recruit *Tregs* by expressing the chemokine CCL20 via the EBV core antigen EBNA1 [35], which allows cancer cells to evade immune surveillance.

In this study, the positive rates of *F. nucleatum* infection and *Treg* enrichment were significantly higher in ESCC tissues than in paracancerous tissues, suggesting that the microenvironment of cancer tissues is more suitable for *F. nucleatum* colonization and *Treg* enrichment. Moreover, there was a significant consistency between *F. nucleatum* infection and *Tregs* infiltration into cancer tissues, suggesting that *F. nucleatum* may provide favorable conditions for long-term colonization by recruiting *Tregs* and suppressing immune responses. At the same time, the massive enrichment of *Tregs* could also weaken the immune response and assist in long-term colonization of *F. nucleatum*. Furthermore, we analyzed the correlation of *F. nucleatum* infection with clinicopathological characteristics of patients with ESCC using the chi-squared test and found that *F. nucleatum* infection was significantly correlated with sex, smoking, and alcohol consumption in patients with ESCC. The majority of *F. nucleatum* infection-positive patients were men who smoked and consumed alcohol, which indicated that long-term smoking and alcohol consumption led to a poor oral and esophageal environment, making patients more susceptible to *F. nucleatum* infection and colonization. The positive rates of *F. nucleatum* infection was significantly higher in hypodifferentiated ESCC tissues than in tissues with medium-high differentiation, suggesting that *F. nucleatum* infection may correlate with tumor malignancy. Meanwhile, *F. nucleatum* infection was significantly correlated with the depth of tumor infiltration, lymph node metastasis, and clinical stage, suggesting that *F. nucleatum* infection may promote the malignant progression of the tumor. This study also found that the degree of tumor differentiation, depth of infiltration, lymph node metastasis, clinical stage, and copositivity for *F. nucleatum* infection were independent risk factors affecting the prognosis of ESCC. The 5 years survival rate and median survival time of patients in the *F. nucleatum* infection-positive group was significantly lower than those in the respective negative groups, suggesting that effective elimination of *F. nucleatum* may prolong the survival of patients with ESCC. Since tumor development is a multifactorial and multi-step evolutionary process, the specific pathogenic mechanism of *F. nucleatum* needs to be further explored, but disrupting the persistent colonization of *F. nucleatum* in the host and effectively inhibiting the massive enrichment of *Tregs* are of great importance to actively and effectively delaying the malignant progression of ESCC and prolonging the survival of patients.

## Data Availability

The original contributions presented in the study are included in the article/supplementary material, further inquiries can be directed to the corresponding author.
